# Multicomponent Synthesis of Luminescent Iminoboronates

**DOI:** 10.3390/molecules25246039

**Published:** 2020-12-21

**Authors:** Samuel Guieu, Cátia I. C. Esteves, João Rocha, Artur M. S. Silva

**Affiliations:** 1 LAQV/REQUIMTE, Department of Chemistry, University of Aveiro, 3810-193 Aveiro, Portugal; catiaiesteves@ua.pt; 2 CICECO-Aveiro Institute of Materials, Department of Chemistry, University of Aveiro, 3810-193 Aveiro, Portugal; rocha@ua.pt

**Keywords:** anthranilic acid, 2-hydroxybenzaldehydes, phenylboronic acid, fluorescence, aggregation-induced emission enhancement

## Abstract

A family of iminoboronates was prepared through a one-pot multicomponent reaction, starting from boronic acid, anthranilic acid, and different salicylaldehydes. Their synthesis was straightforward and the complexes were obtained in good to excellent yields. Their photophysical properties were assessed in a diluted solution, and the complexes proved to be faintly luminescent. These chelates demonstrated remarkable Aggregation-Induced Emission Enhancement, which was rationalized using crystal structures.

## 1. Introduction

Boron complexes based on N and O donor ligands have been widely studied due to their ease of preparation and versatility [1]. In terms of luminescent properties, boron complexes are dominated by boron dipyrromethenes (BODIPYs) [2], but recently, boron diketonates [3,4,5] and ketoiminates [6] have emerged as promising alternatives. Moreover, boranils appeared as more versatile [7], being very easily prepared from building blocks that can be modified to tune the properties of the final complex [8,9]. Boron complexes usually suffer a quenching of their emission upon aggregation, which limits their use in luminescent materials. However, they can present the opposite behavior, aggregation-induced emission enhancement (AIEE) [10], when adequately substituted with aromatic rotating groups [11,12,13,14,15]. In these cases, the emission intensity increases upon aggregation, due to the restriction of molecular motions and absence of excimer formations. In these complexes, the ligand is usually bidentate, and the boron bears two fluorine or aromatic substituents. The use of a tridentate ligand is less explored and mainly relies on the use of boronic acid or ester as precursor of the complex.

Boronic acids are widely used for the fluorescent sensing of carbohydrates [16], but rarely used for the synthesis of fluorescent dyes [17,18,19,20,21]. However, they have been used as building blocks in the synthesis of analogs of natural products [22], and advantage has been taken of their reversible bond formations in the complexes, allowing the synthesis to be performed through one-pot multicomponent reactions [23]. In these cases, the multicomponent reaction usually involves the formation of an imine between two building blocks also bearing hydroxy or carboxy groups, and the complexation of the boron into the formed tridentate ligand formed [24]. Here, we report the one-pot multicomponent synthesis of iminoboronates, starting from readily available building blocks, and the study of their photophysical properties.

## 2. Results and Discussion

### 2.1. Synthesis

**Scheme 1:**
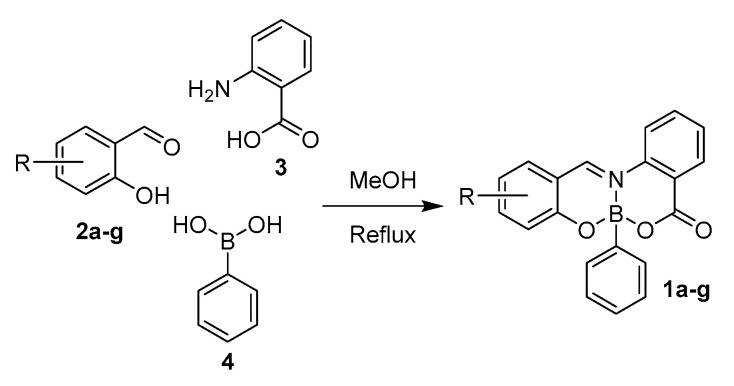
Multicomponent synthesis of the boron complexes.

The boron complexes **1a**–**g** were readily obtained in one step (Scheme 1). The imine was formed in situ by the condensation of the appropriate substituted salicylaldehydes **2a**–**g** with anthranilic acid **3** and underwent a double condensation with the phenylboronic acid **4** to form the boron complex in good to excellent yields. This strategy was largely inspired by previously reported procedures but avoids the use of highly toxic carbon tetrachloride as a solvent and uses conventional heating instead of microwave irradiation [25].

**Figure 1:**
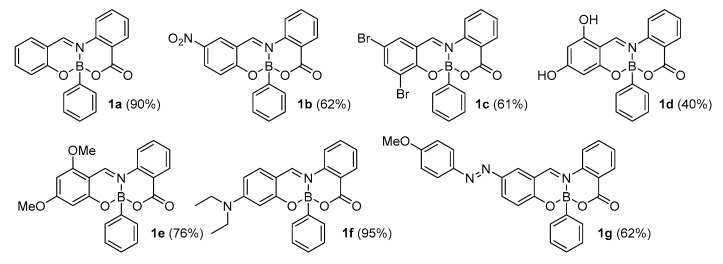
Boron complexes obtained through the one-pot multicomponent reaction, and corresponding yield.

This simple and modular approach allowed the synthesis of a family of boron complexes (Figure 1) with various substituents. The substituents did not seem to influence the yield of the reaction, nor its selectivity, as both electron donating (hydroxyl, ether, amino) and electron withdrawing (nitro, bromo) groups were able to be introduced on the salicylaldehyde moieties. Moreover, hydroxy groups can be present and do not seem to disrupt the boron complexation, the lower yield obtained in the case of **1d** being ascribed to its incomplete precipitation in methanol. Salicylaldehyde **2g**, bearing a diazo substituent, was also used to increase the conjugation of the backbone and therefore modify the color of the product. All compounds were fully characterized by ^1^H and ^13^C NMR, and MS (See Supplementary Materials). The complexes bore a chiral boron center and were obtained as a racemic mixture. The separation of the enantiomers was not attempted, even if it may be possible [20].

### 2.2. Photophysical Properties

**Figure 2:**
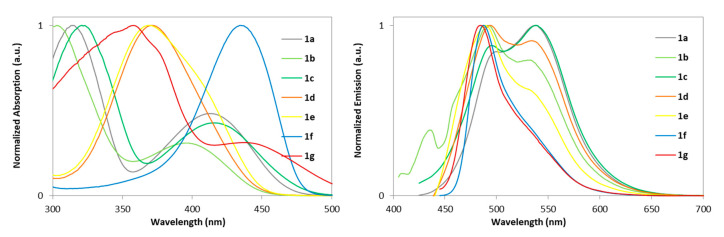
Absorption (**left**) and emission (**right**) spectra of boron complexes in THF solutions.

Boron complexes often present interesting photophysical properties, such as high absorption coefficients and quantum yields, but iminoboronates have only been described as faintly luminescent in solution [25]. Therefore, the absorption and emission properties of the prepared boron complexes were studied in THF solutions. The absorption spectra present one or two bands (Figure 2, left), the main band being between 360 and 450 nm (Table 1). As expected, the complexes **1f**,**g**, bearing a strong electron donating substituent or with an extended conjugation, presented a bathochromic shift of the main band. The effect of the other substituents is more difficult to assess.

All compounds were emissive in a dilute solution, with low quantum yields (Table 1). The emission profile is composed of two bands (Figure 2, right), and the relative intensity depends on the substituents. The intensity of the band at longer wavelengths seemed to decrease when substituents were introduced, but the trend was not obvious. This band almost disappeared when the dyes bore strong electron donating substituents (**1e**,**f**) and when the conjugation increased (**1g**). Overall, the dyes were only faintly emissive, with quantum yields ranging from 0.1% to 6.0%, which is in accordance with previously reported observations [20].

**Figure 3:**
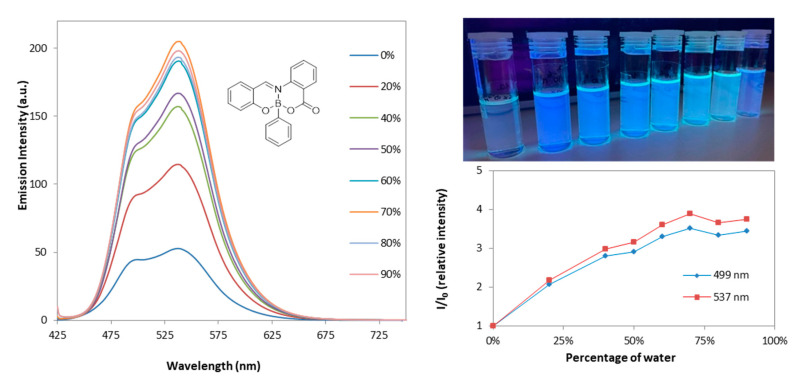
Emission spectra of boron complex **1a** in THF–water solutions (**left**); photographs of the solutions/suspensions under UV light (365 nm) and relative emission intensities as a function of the percentage of water (**right**).

As the compounds **1a**–**g** seemed luminescent in their powder or crystalline forms, when irradiated with a hand-held UV-lamp, we performed a classical AIEE test. The emission spectra of all compounds were recorded in THF-water mixed solvents at the same concentration and with different proportions of water (See Supplementary Materials). Boron complex **1a** exhibits a classical AIEE behavior (Figure 3): when water is added, the compound precipitates and the intensity of the emission increases. When more water is added, the quantity of solid in the suspension increases, and so does the emission intensity up to 4 times the initial intensity. The emission wavelength does not change between solution and aggregates, ruling out the formation of excimers. Unfortunately, all the other complexes **1b**–**g** displayed a different behavior: either they presented a quenching of their emission when they precipitated (**1c**,**f**,**g**), or a shift in their emission wavelength with no clear trend, probably due to the formation of excimers/exciplexes in the aggregate form.

### 2.3. Crystal Structures

Single crystals suitable for X-ray diffraction were successfully grown for compounds **1a**,**d**,**e**, by the slow evaporation of saturated solutions in dichloromethane-methanol. The crystal structure of **1a** has already been published [25], but was determined again for consistency.

**Figure 4:**
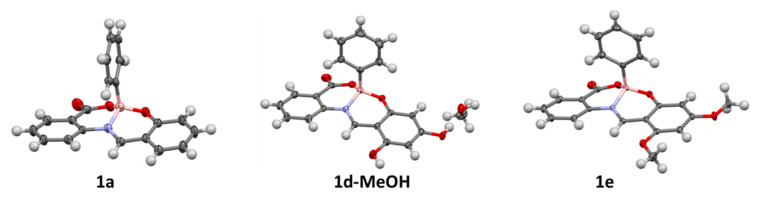
Asymmetric unit of boron complexes **1a**, **1d-MeOH**, and **1e** as revealed by single crystal X-ray diffraction. Thermal ellipsoids are shown at the 50% probability level; hydrogen atoms are shown with an arbitrary radius (0.30 Å). C, grey; N, blue; O, red; B, pink; H, white.

As already mentioned, the complexes were obtained as racemic mixtures, and crystallized as such. They crystallized in centrosymmetric groups, and their asymmetric unit was composed of one enantiomer, the other being generated by symmetry (Figure 4 and Supplementary Materials). All bond lengths and angles are in normal range [27]. Complex **1d** crystallized together with one molecule of methanol, accepting one hydrogen bond from a phenol hydroxy group and donating one hydrogen bond to the carboxylic oxygen linked to the boron center.

The ligands adopted a non-planar geometry to accommodate the tetrahedral boron center. The dihedral angle N-B-C-C between the phenyl of the boronic acid and the ligand was the main difference between the complexes: −17.17° for **1a**, −57.48° for **1d** and −110.11° for **1e**. In the crystal packing, **1a** arranged into linear chains through C-H···O hydrogen bonds, and there were no close contacts between the chains, which restrained the molecular motions without introducing the formation of excimers, thus rationalizing the AIEE behavior observed. By contrast, both **1d** and **1e** arranged into tridimensional networks involving multiple close contacts, which probably favored the formation of excimers and exciplexes, resulting in a quenching of the emission in the solid state.

## 3. Materials and Methods

General procedure for the synthesis of the complexes: the appropriate salicylaldehyde derivative (1 equiv, 1 mmol) was dissolved in MeOH (20 mL). Anthranilic acid (1 equiv, 1 mmol) was added, followed by phenylboronic acid (1 equiv, 1 mmol), and the reaction mixture was refluxed for 12 h. After cooling down to room temperature, the solid was collected by filtration, washed with MeOH, and dried in air. When necessary, the product was further purified by silica gel flash column chromatography. The boron complexes were obtained as colored solids in 40–95% yield.

### 3.1. 7-Phenyl-5H,7H-7λ^4^,14λ^4^-benzo[d]benzo[5,6][1,3,2]oxazaborinino[2,3-b][1,3,2]oxazaborinin-5-one ***1a***

Salicylaldehyde (1 equiv, 1 mmol, 122 mg) was dissolved in MeOH (20 mL). Anthranilic acid (1 equiv, 1 mmol, 137 mg) was added, followed by phenylboronic acid (1 equiv, 1 mmol, 122 mg), and the reaction mixture was refluxed for 12 h. After cooling down to room temperature, the solid was collected by filtration, washed with MeOH, and dried in air. The product was obtained as a yellow solid (295 mg, 90% yield) without further purification. The compound gave single crystals suitable for X-ray diffraction by slow evaporation from a saturated solution in DCM/MeOH.

m.p. 259–261 °C. ^1^H NMR (300.13 MHz, CDCl_3_, 25 °C): δ = 8.70 (s, 1H, C*H*N), 8.27 (dd, ^3^*J*_H-H_ 7.8, ^4^*J*_H-H_ 1.2 Hz, 1H, aromatic C*H*), 7.69–7.59 (m, 3H, aromatic C*H*), 7.54–7.49 (m, 2H, aromatic C*H*), 7.29–7.26 (m, 2H, aromatic C*H*), 7.14–7.08 (m, 4H, aromatic C*H*), 6.99 (ddd, ^3^*J*_H-H_ 8.1, ^3^*J*_H-H_ 8.1, ^4^*J*_H-H_ 1.2 Hz, 1H, aromatic C*H*). ^13^C NMR (75 MHz, CDCl_3_, 25 °C): δ = 161.8 (*C*=O), 160.3 (*C*=N), 158.3 (2C, *C*-O, *C*-N), 140.7 (*C*-B), 139.8 (*C*quat), 134.4 (*C*-H), 132.9 (*C*-H), 132.3 (*C*-H), 130.6 (*C*-H), 130.0 (*C*-H), 128.1 (*C*-H), 127.7 (*C*-H), 125.1 (*C*-H), 120.5 (*C*-H), 120.3 (*C*-H), 117.7 (*C*-H), 116.1 (*C*quat).

### 3.2. 11-Nitro-7-phenyl-5H,7H-7λ^4^,14λ^4^-benzo[d]benzo[5,6][1,3,2]oxazaborinino[2,3-b][1,3,2]oxazaborinin-5-one ***1b***

*p*-Nitrosalicylaldehyde (1 equiv, 1 mmol, 167 mg) was dissolved in MeOH (20 mL). Anthranilic acid (1 equiv, 1 mmol, 137 mg) was added, followed by phenylboronic acid (1 equiv, 1 mmol, 122 mg), and the reaction mixture was refluxed for 4 h. After cooling down to room temperature, the solid was collected by filtration, washed with MeOH, and dried in air. The product was obtained as a yellow solid (230 mg, 62% yield) without further purification.

m.p. 353–355 °C. ^1^H NMR (300.13 MHz, Acetone-*d*_6_, 25 °C): d = 9.81 (s, 1H, C*H*N), 8.85 (d, ^4^*J*_H-H_ 3.0 Hz, 1H, aromatic C*H*), 8.53 (dd, ^3^*J*_H-H_ 9.0, ^4^*J*_H-H_ 3.0 Hz, 1H, aromatic C*H*), 8.17–8.14 (m, 2H, aromatic C*H*), 7.85 (ddd, ^3^*J*_H-H_ 5.7, ^3^*J*_H-H_ 8.1, ^4^*J*_H-H_ 1.5 Hz, 1H, aromatic C*H*), 7.65 (ddd, ^3^*J*_H-H_ 7.5, ^3^*J*_H-H_ 7.5, ^4^*J*_H-H_ 0.9 Hz, 1H, aromatic C*H*), 7.31–7.27 (m, 2H, aromatic C*H*), 7.22 (d, ^3^*J*_H-H_ 9.0 Hz, 1H, aromatic C*H*), 7.13–7.09 (m, 3H, aromatic C*H*). ^13^C NMR (75 MHz, Acetone-*d_6_*, 25 °C): d = 162.2 (*C*=O), 161.5 (*C*=N), 157.4 (*C*-O), 153.1 (*C*-N), 140.6 (*C*-B), 135.4 (*C*quat), 134.5 (*C*-H), 132.0 (*C*-H), 131.3 (*C*-H), 131.2 (2C, *C*-H), 131.0 (*C*-H), 128.8 (*C*-H), 128.4 (2C, *C*-H, *C*quat), 125.9 (*C*quat), 121.1 (*C*-H), 120.2 (*C*-H). ESI^+^-MS *m*/*z* = 373.1 [M + H]^+^, 395.1 [M + Na]^+^; HRMS-ESI^+^ *m*/*z* for [C_20_H_13_O_5_N_2_B + H]^+^ calcd 373.0996, found 373.0981; HRMS-ESI^+^ *m*/*z* for [C_20_H_13_O_5_N_2_B + Na]^+^ calcd 395.0815, found 395.0809.

### 3.3. 9,11-Dibromo-7-phenyl-5H,7H-7λ^4^,14λ^4^-benzo[d]benzo[5,6][1,3,2]oxazaborinino[2,3-b][1,3,2]oxazaborinin-5-one ***1c***

4,6-Dibromosalicylaldehyde (1 equiv, 0.25 mmol, 70 mg) was dissolved in MeOH (20 mL). Anthranilic acid (1 equiv, 0.25 mmol, 34 mg) was added, followed by phenylboronic acid (1 equiv, 0.25 mmol, 31 mg), and the reaction mixture was refluxed for 2 h. After cooling down to room temperature, the solid was collected by filtration, washed with MeOH, and dried in air. The product was obtained as a yellow solid (74 mg, 61% yield) without further purification.

m.p. 353–355 °C. ^1^H NMR (300.13 MHz, Acetone-*d_6_*, 25 °C): d = 9.60 (s, 1H, C*H*N), 8.17–8.12 (m, 3H, aromatic C*H*), 8.05 (bs, 1H, aromatic C*H*), 7.85 (dd, ^3^*J*_H-H_ 7.7, ^3^*J*_H-H_ 7.7 Hz, 1H, aromatic C*H*), 7.66 (dd, ^3^*J*_H-H_ 7.4, ^3^*J*_H-H_ 7.4 Hz, 1H, aromatic C*H*), 7.25 (bs, 2H, aromatic C*H*), 7.10 (br s, 3H, aromatic C*H*). ^13^C NMR (75 MHz, Acetone-*d_6_*, 25 °C): d = 161.4 (*C*=O), 161.3 (*C*=N), 155.9 (2C, *C*-O, *C*-N), 144.2 (*C*-H), 140.5 (*C*-B), 135.9 (*C*-H), 135.4 (*C*-H), 132.0 (*C*-H), 131.3 (*C*-H), 128.6 (2C, *C*-H, *C*-H), 128.3 (*C*-H), 125.8 (*C*-H), 120.1 (*C*quat), 119.6 (*C*quat), 114.6 (*C*-Br), 111.3 (*C*-Br). ESI^+^-MS *m*/*z* = 483.9, 485.9, 487.9 [M + H]^+^, 505.9, 507.9, 507.9 [M + Na]^+^; HRMS-ESI^+^ *m*/*z* for [C_20_H_12_O_3_NBBr_2_ + H]^+^ calcd 485.9335, found 485.9319.

### 3.4. 10,12-Dihydroxy-7-phenyl-5H,7H-7λ^4^,14λ^4^-benzo[d]benzo[5,6][1,3,2]oxazaborinino[2,3-b][1,3,2]oxazaborinin-5-one ***1d***

2,4,6-Trihydroxybenzaldehyde (1 equiv, 0.5 mmol, 77 mg) was dissolved in MeOH (20 mL). Anthranilic acid (1 equiv, 0.5 mmol, 69 mg) was added, followed by phenylboronic acid (1 equiv, 0.5 mmol, 61 mg), and the reaction mixture was refluxed for 2 h. After cooling down to room temperature, the solid is collected by filtration, washed with MeOH, and dried in air. After silica gel flash column chromatography (eluent: DCM/MeOH, 90/10), the product was obtained as a yellow solid (72 mg, 40% yield). The compound gave single crystals suitable for X-ray diffraction by slow evaporation from a saturated solution in MeOH.

m.p. > 350 °C. ^1^H NMR (300.13 MHz, Acetone-*d*_6_, 25 °C): d = 9.10 (s, 1H, C*H*N), 8.02 (d, ^3^*J*_H-H_ 8.0 Hz, 1H, aromatic C*H*), 7.96 (d, ^3^*J*_H-H_ 8.0 Hz, 1H, aromatic C*H*), 7.71 (dd, ^3^*J*_H-H_ 7.8, ^3^*J*_H-H_ 7.8 Hz, 1H, aromatic C*H*), 7.42 (dd, ^3^*J*_H-H_ 7.2, ^3^*J*_H-H_ 7.2 Hz, 1H, aromatic C*H*), 7.16–7.06 (m, 5H, aromatic C*H*), 5.92 (d, ^4^*J*_H-H_ 1.8 Hz, 1H, aromatic C*H*), 5.80 (d, ^4^*J*_H-H_ 1.8 Hz, 1H, aromatic C*H*). ^13^C NMR (75 MHz, Acetone-*d_6_*, 25 °C): d = 170.6 (*C*=O), 162.7 (*C*=N), 161.8 (*C*-O), 161.0 (*C*-O), 152.5 (*C*-O), 150.7 (*C*-N), 140.5 (*C*-B), 134.5 (*C*-H), 130.4 (*C*-H), 130.2 (*C*-H), 127.6 (*C*-H), 127.3 (*C*-H), 127.2 (*C*-H), 122.7 (*C*-H), 118.5 (*C*quat), 101.8 (*C*quat), 95.8 (*C*-H), 94.6 (*C*-H). ESI^+^-MS *m*/*z* = 360.1 [M + H]^+^, 382.1 [M + Na]^+^; HRMS-ESI^+^ *m*/*z* for [C_20_H_14_O_5_NB + H]^+^ calcd 360.1043, found 360.1035.

### 3.5. 10,12-Dimethoxy-7-phenyl-5H,7H-7λ^4^,14λ^4^-benzo[d]benzo[5,6][1,3,2]oxazaborinino[2,3-b][1,3,2]oxazaborinin-5-one ***1e***

3,5-Dimethoxysalicylaldehyde (1 equiv, 1 mmol, 182 mg) was dissolved in MeOH (20 mL). Anthranilic acid (1 equiv, 1 mmol, 137 mg) was added, followed by phenylboronic acid (1 equiv, 1 mmol, 122 mg), and the reaction mixture was refluxed for 4 h. After cooling down to room temperature, the solid was collected by filtration, washed with MeOH, and dried in air. The product was obtained as a yellow solid (293 mg, 76% yield) without further purification.

m.p. 251–253 °C. ^1^H NMR (300.13 MHz, Acetone-*d*_6_, 25 °C): δ = 9.24 (s, 1H, C*H*N), 8.07 (dd, ^3^*J*_H-H_ 7.8, ^4^*J*_H-H_ 1.5 Hz, 1H, aromatic C*H*), 8.04 (d, ^3^*J*_H-H_ 8.4 Hz, 1H, aromatic C*H*), 7.73 (ddd, ^3^*J*_H-H_ 7.2, ^3^*J*_H-H_ 8.1, ^4^*J*_H-H_ 1.5 Hz, 1H, aromatic C*H*), 7.47 (ddd, ^3^*J*_H-H_ 7.8, ^3^*J*_H-H_ 7.8, ^4^*J*_H-H_ 1.0 Hz, 1H, aromatic C*H*), 7.29–7.26 (m, 2H, aromatic C*H*), 7.10–7.07 (m, 3H, aromatic C*H*), 6.14 (s, 2H, aromatic C*H*), 3.94 (s, 3H, OC*H*_3_), 4.00 (s, 3H, OC*H*_3_). ^13^C NMR (75 MHz, Acetone-*d*_6_, 25 °C): δ = 172.6 (*C*-OCH_3_), 163.5 (*C*-OCH_3_), 153.8 (*C*=N), 150.8 (*C*=O), 146.4 (*C*-O), 142.0 (*C*-N), 141.7 (*C*-B), 135.0 (*C*-H), 131.8 (*C*-H), 131.4 (*C*-H), 128.9 (*C*-H), 128.1 (*C*-H), 125.1 (*C*-H), 119.2 (*C*-H), 108.7 (*C*quat), 103.7 (*C*quat), 95.5 (*C*-H), 92.2 (*C*-H), 56.9 (O*C*H_3_), 56.8 (O*C*H_3_). ESI^+^-MS *m*/*z* = 388.1 [M + H]^+^, 410.1 [M + Na]^+^; HRMS-ESI^+^ *m*/*z* for [C_22_H_18_O_5_NB + H]^+^ calcd 388.1356, found 388.1351.

### 3.6. 10-(Diethylamino)-7-phenyl-5H,7H-7λ^4^,14λ^4^-benzo[d]benzo[5,6][1,3,2]oxazaborinino[2,3-b][1,3,2]oxazaborinin-5-one ***1f***

4-Diethylaminosalicylaldehyde (1 equiv, 0.5 mmol, 96 mg) was dissolved in MeOH (10 mL). Anthranilic acid (1 equiv, 0.5 mmol, 69 mg) was added, followed by phenylboronic acid (1 equiv, 0.5 mmol, 61 mg), and the reaction mixture was refluxed for 1 h. After cooling down to room temperature, the solid was collected by filtration, washed with MeOH, and dried in air. The product was obtained as a yellow solid (190 mg, 95% yield) without further purification.

m.p. 284–286 °C. ^1^H NMR (300.13 MHz, Acetone-*d*_6_, 25 °C): d = 8.98 (s, 1H, C*H*N), 8.04 (dd, ^3^*J*_H-H_ 7.8, ^4^*J*_H-H_ 1.2 Hz, 1H, aromatic C*H*), 7.90 (d, ^3^*J*_H-H_ 9.0 Hz, 1H, aromatic C*H*), 7.66 (ddd, ^3^*J*_H-H_ 7.2, ^3^*J*_H-H_ 8.1, ^4^*J*_H-H_ 1.5 Hz, 1H, aromatic C*H*), 7.49 (d, ^3^*J*_H-H_ 9.3 Hz, 1H, aromatic C*H*), 7.36 (ddd, ^3^*J*_H-H_ 7.8, ^3^*J*_H-H_ 7.8, ^4^*J*_H-H_ 1.0 Hz, 1H, aromatic C*H*), 7.29–7.26 (m, 2H, aromatic C*H*), 7.10–7.03 (m, 3H, aromatic C*H*), 6.54 (dd, ^3^*J*_H-H_ 9.3, ^4^*J*_H-H_ 2.4 Hz, 1H, aromatic C*H*), 6.13 (d, ^4^*J*_H-H_ 2.1 Hz, 1H, aromatic C*H*), 3.57 (q, ^3^*J*_H-H_ 7.2 Hz, 4H, NC*H*_2_), 1.23 (t, ^3^*J*_H-H_ 7.2 Hz, 6H, C*H*_3_). ^13^C NMR (75 MHz, Acetone-*d_6_*, 25 °C): d = 162.9 (*C*=O), 162.8 (*C*-O), 158.2 (*C*-N), 154.8 (*C*=N), 142.3 (*C*-N), 141.7 (*C*-B), 136.2 (*C*-H), 134.7 (*C*-H), 131.8 (*C*-H), 131.4 (*C*-H), 128.0 (*C*-H), 127.8 (*C*-H), 127.5 (*C*-H), 124.6 (*C*quat), 118.2 (*C*-H), 108.5 (*C*quat), 107.8 (*C*-H), 98.4 (*C*-H), 45.7 (*C*H_2_CH_3_), 13.0 (CH_2_*C*H_3_). ESI^+^-MS *m*/*z* = 399.2 [M + H]^+^, 421.2 [M + Na]^+^; HRMS-ESI^+^ *m*/*z* for [C_24_H_23_O_3_N_2_B + H]^+^ calcd 399.1880, found 399.1869; HRMS-ESI^+^ *m*/*z* for [C_24_H_23_O_3_N_2_B + Na]^+^ calcd 421.1699, found 421.1689.

### 3.7. (E)-11-[(4-Methoxyphenyl)diazenyl]-7-phenyl-5H,7H-7λ^4^,14λ^4^-benzo[d]benzo[5,6][1,3,2]oxazaborinino[2,3-b][1,3,2]oxazaborinin-5-one ***1g***

(*E*)-2-Hydroxy-5-[(4-methoxyphenyl)diazenyl]benzaldehyde (1 equiv, 0.25 mmol, 64 mg) was dissolved in MeOH (20 mL). Anthranilic acid (1 equiv, 0.25 mmol, 34 mg) was added, followed by phenylboronic acid (1 equiv, 0.25 mmol, 31 mg), and the reaction mixture was refluxed for 5 h. After cooling down to room temperature, the solid was collected by filtration, washed with MeOH, and dried in air. The product was obtained as a yellow solid (71 mg, 62% yield) without further purification.

m.p. 282–284 °C. ^1^H NMR (300.13 MHz, Acetone-*d*_6_, 25 °C): δ = 9.74 (s, 1H, C*H*N), 8.39 (d, ^4^*J*_H-H_ 2.5 Hz, 1H, aromatic C*H*), 8.28 (dd, ^3^*J*_H-H_ 9.0, ^4^*J*_H-H_ 2.5 Hz, 1H, aromatic C*H*), 8.20–8.13 (m, 2H, aromatic C*H*), 7.92 (d, ^3^*J*_H-H_ 9.0 Hz, 2H, aromatic C*H*), 7.83 (ddd, ^3^*J*_H-H_ 8.2, ^3^*J*_H-H_ 7.4, ^4^*J*_H-H_ 1.6 Hz, 1H, aromatic C*H*), 7.62 (ddd, ^3^*J*_H-H_ 7.6, ^3^*J*_H-H_ 7.6, ^4^*J*_H-H_ 1.1 Hz, 1H, aromatic C*H*), 7.34–7.28 (m, 2H, aromatic C*H*), 7.19 (d, ^3^*J*_H-H_ 9.0 Hz, 1H, aromatic C*H*), 7.16–7.08 (m, 5H, aromatic C*H*), 3.92 (s, 3H, OC*H*_3_). ^13^C NMR (75 MHz, Acetone-*d_6_*, 25 °C): δ = 163.3 (*C*=O), 162.3 (*C*=N), 162.1 (*C*-O), 161.8 (*C*-O), 147.5 (*C*-N), 146.7 (*C*-N), 140.9 (*C*-B), 135.3 (*C*-N), 133.7 (*C*-H), 131.9 (*C*-H), 131.3 (*C*-H), 130.7 (*C*-H), 129.6 (*C*-H), 128.5 (*C*-H), 128.3 (*C*-H), 125.8 (*C*quat), 125.4 (*C*-H), 123.7 (*C*quat), 120.9 (*C*-H), 120.1 (*C*-H), 115.3 (*C*-H), 114.9 (*C*-H), 56.1 (O*C*H_3_). ESI^+^-MS *m*/*z* = 462.2 [M + H]^+^, 484.1 [M + Na]^+^; HRMS-ESI^+^ *m*/*z* for [C_27_H_20_O_4_N_3_B + H]^+^ calcd 462.1625, found 462.1609; HRMS-ESI^+^ *m*/*z* for [C_27_H_20_O_4_N_3_B + Na]^+^ calcd 484.1445, found 484.1429.

## 4. Conclusions

A straightforward one-pot multicomponent reaction was implemented, allowing the rapid preparation of boron complexes based on phenylboronic acid. This versatile methodology was compatible with a wide range of derivatives, and the complexes were decorated with electron donating or withdrawing substituents. They proved to be faintly luminescent in a dilute solution, but the quantum yield increased when a strongly electron donating substituent was adequately placed. The parent compound demonstrated aggregation-induced emission enhancement, but unfortunately, the emission intensity of the other complexes was quenched in the solid state, probably due to the formation of excimers. Overall, this study should open the way to the design and synthesis of other boron complexes, which may find applications as luminescent materials or probes.

## Figures and Tables

**Table 1:** Absorption and emission properties of the complexes **1a**–**g**.

Complex	THF Solution			
	λ_max abs_	Log ε	λ_max em_^1^	Φ^2^ (%)
**1a**	412	3.92	537	0.7
**1b**	396	3.90	491	0.2
**1c**	415	3.80	538	0.4
**1d**	371	4.44	494	0.5
**1e**	368	4.43	489	0.3
**1f**	434	4.72	487	6.0
**1g**	445	3.94	480	0.1

^1^ Upon excitation at the maximum of absorption. ^2^ Determined by comparison with fluorescein (quantum yield of 0.90 at an excitation of 470 nm in a solution of NaOH 0.01 M in water) [26].

## Supplementary Materials

The following are available online: ^1^H and ^13^C NMR spectra, absorption, and emission spectra. Details of the crystal data collection, solution, and refinement of compounds **1a**, **1d-MeOH**, and **1e**. Crystal structures in CIF format.
