# Supplementary material for: Multicomponent Synthesis of Luminescent Iminoboronates
Source: Molecules. 2020 Dec 21;25(24):6039. doi: 10.3390/molecules25246039 (PMC7766465; doi:10.3390/molecules25246039)
Supplement: Supplementary file 1 [file molecules-25-06039-s001.zip › cif deposition.pdf]

CCDC Depository Request

CCDC Deposit <deposit_reply@ccdc.cam.ac.uk>

mer. 25/11/2020 14:38

À :Samuel Guieu <sguieu@ua.pt>;

Dear Depositor,

Thank you for depositing your crystal structure(s) via the joint CCDC/FIZ Karlsruhe deposition service.

The data have been assigned the following deposition numbers which can either be quoted as CCDC Numbers or CSD Numbers. A CCDC Number is usually quoted for an organic or metal-organic structure, whereas a CSD Number is usually quoted for an inorganic structure.

CCDC XXXXXXX-YYYYYYY (generally used for organic and metal-organic structures)

CSD XXXXXXX-YYYYYYY (generally used for inorganic structures)

Deposition Number 2046453-2046455

Summary of Data - Deposition Number 2046453

Compound Name:
Data Block Name: data_samuel_sg696_0m
Unit Cell Parameters: a 28.300(2) b 7.6129(7) c 17.6359(14) C2/c
----

Summary of Data - Deposition Number 2046454

Compound Name:
Data Block Name: data_shelx
Unit Cell Parameters: a 11.1926(5) b 10.5497(5) c 15.8352(8) P21/c
----

Summary of Data - Deposition Number 2046455

Compound Name:
Data Block Name: data_shelx

Unit Cell Parameters: a 8.8820(6) b 11.6380(7) c 18.0177(10) P21/c
----

After publication your data will be made available through our joint Access Structures service. In addition, organic and metal-organic experimental structures will be curated into the Cambridge Structural Database and inorganic experimental structures will be curated into the Inorganic Crystal Structure Database.

If you selected “Publish in a Database” your data will be immediately published through our joint Access Structures service.

Please note, if any of these structures are not published within one year from today and we cannot contact you to discuss the matter, then we may publish the data directly through the CSD as a *CSD Communication* or the ICSD as an *ICSD Communication*.

If we have any queries relating to the data then we may contact you later.

Kind Regards,

The CCDC and FIZ Karlsruhe Deposition Teams

Email: deposit@ccdc.cam.ac.uk
The Cambridge Crystallographic Data Centre
https://www.ccdc.cam.ac.uk
For more information about CSD Communications see:
https://www.ccdc.cam.ac.uk/Community/Depositastructure/CSDCommunications/
FIZ Karlsruhe
www.fiz-karlsruhe.de

The CCDC and FIZ Karlsruhe are delighted to be working together on shared deposition and access services for crystallographic data across all domains of chemistry

More details can be found in our press release: https://www.ccdc.cam.ac.uk/News/List/2018-07-new-joint-services/
