# Supplementary material for: Multicomponent Synthesis of Luminescent Iminoboronates
Source: Molecules. 2020 Dec 21;25(24):6039. doi: 10.3390/molecules25246039 (PMC7766465; doi:10.3390/molecules25246039)
Supplement: Supplementary file 1 [file molecules-25-06039-s001.zip › Molecules SI.docx]

**Multicomponent synthesis of luminescent boron complexes**

Samuel Guieu ^1,2,^*, Cátia I. C. Esteves ^1^, João Rocha ^2^ and Artur M. S. Silva ^1,^*

^1^ LAQV / REQUIMTE, University of Aveiro, Department of Chemistry, 3810-193 Aveiro, Portugal; [sguieu@ua.pt](mailto:sguieu@ua.pt)

^2^ CICECO-Aveiro Institute of Materials, University of Aveiro, Department of Chemistry, 3810-193 Aveiro, Portugal; [rocha@ua.pt](mailto:rocha@ua.pt)

Correspondence: [sguieu@ua.pt](mailto:sguieu@ua.pt), [artur.silva@ua.pt](mailto:artur.silva@ua.pt)

[1. Synthesis of the complexes 2](#_Toc57807824)

[1.1. General 2](#_Toc57807825)

[1.2. General procedure 2](#_Toc57807826)

[1.3. Synthesis 2](#_Toc57807827)

[2. NMR spectra 6](#_Toc57807828)

[3. Details of the single-crystals XRD 13](#_Toc57807829)

[4. Absorption and emission spectra 14](#_Toc57807830)

[5. Bibliography 19](#_Toc57807831)

# Synthesis of the complexes

## General

All reagents were purchased from Sigma-Aldrich and used without any further purification. ^1^H and ^13^C NMR spectra were recorded on Bruker 300 [300.13 MHz (^1^H), 75.47 MHz (^13^C)]. Unequivocal ^13^C assignments were made on the basis of 2D HSQC (^1^H/^13^C) and HMBC experiments. Chemical shifts (δ) are reported in parts per million (ppm) relative to TMS (δ = 0), and the values of coupling constants (*J*) are given in Hertz (Hz). High-resolution mass spectra (HRMS-ESI^+^) were recorded on an LTQ Orbitrap^TM^ XL hybrid mass spectrometer (Thermo Fischer Scientific, Bremen, Germany) controlled by LTQ Tune Plus 2.5.5 and Xcalibur 2.1.0. The capillary voltage of the electrospray ionization source (ESI) was set to 3.1 kV. Melting points were determined on a BUCHI Melting point apparatus and are uncorrected. The ultraviolet-visible (UV-Vis) spectra were obtained on a Shimadzu UV-2501 PC spectrophotometer (1 cm path length quartz cell). The excitation and emission spectra were recorded on a Jobin Yvon FluoroMax-3 spectrofluorometer and a JASCO spectrofluorometer. Fluorescence quantum yields φF were determined using fluorescein in 0.1 M NaOH water solution as a fluorescence standard.

## General procedure

The appropriate salicylaldehyde derivative (1 equiv, 1 mmol) was dissolved in MeOH (20 mL). Anthranilic acid (1 equiv, 1 mmol) was added, followed by phenylboronic acid (1 equiv, 1 mmol), and the reaction mixture was refluxed for 12 h. After cooling down to room temperature, the solid was collected by filtration, washed with MeOH, and dried in air. If necessary, silica gel flash column chromatography was used to purify the product.

## Synthesis

7-Phenyl-5*H*,7*H*-7λ^4^,14λ^4^-benzo[*d*]benzo[5,6][1,3,2]oxazaborinino[2,3-*b*][1,3,2]oxazaborinin-5-one **1a ^[[1]](#endnote-1)^**

Salicylaldehyde (1 equiv, 1 mmol, 122 mg) was dissolved in MeOH (20 mL). Anthranilic acid (1 equiv, 1 mmol, 137 mg) was added, followed by phenylboronic acid (1 equiv, 1 mmol, 122 mg), and the reaction mixture was refluxed for 12 h. After cooling down to room temperature, the solid was collected by filtration, washed with MeOH, and dried in air. The product was obtained as a yellow solid (295 mg, 90% yield) without further purification. The compound gave single crystals suitable for X-ray diffraction by slow evaporation from a saturated solution in DCM/MeOH.

mp 259-261 °C. ^1^H NMR (300.13 MHz, CDCl_3_, 25 ºC): δ = 8.70 (s, 1H, C*H*N), 8.27 (dd, ^3^*J*_H-H_ 7.8, ^4^*J*_H-H_ 1.2 Hz, 1H, aromatic C*H*), 7.69-7.59 (m, 3H, aromatic C*H*), 7.54-7.49 (m, 2H, aromatic C*H*), 7.29-7.26 (m, 2H, aromatic C*H*), 7.14-7.08 (m, 4H, aromatic C*H*), 6.99 (ddd, ^3^*J*_H-H_ 8.1, ^3^*J*_H-H_ 8.1, ^4^*J*_H-H_ 1.2 Hz, 1H, aromatic C*H*). ^13^C NMR (75 MHz, CDCl_3_, 25ºC): δ = 161.8 (*C*=O), 160.3 (*C*=N), 158.3 (2C, *C*-O, *C*-N), 140.7 (*C*-B), 139.8 (*C*quat), 134.4 (*C*-H), 132.9 (*C*-H), 132.3 (*C*-H), 130.6 (*C*-H), 130.0 (*C*-H), 128.1 (*C*-H), 127.7 (*C*-H), 125.1 (*C*-H), 120.5 (*C*-H), 120.3 (*C*-H), 117.7 (*C*-H), 116.1 (*C*quat).

11-Nitro-7-phenyl-5*H*,7*H*-7λ^4^,14λ^4^-benzo[*d*]benzo[5,6][1,3,2]oxazaborinino[2,3-*b*][1,3,2]oxazaborinin-5-one **1b**

*p*-Nitrosalicylaldehyde (1 equiv, 1 mmol, 167 mg) was dissolved in MeOH (20 mL). Anthranilic acid (1 equiv, 1 mmol, 137 mg) was added, followed by phenylboronic acid (1 equiv, 1 mmol, 122 mg), and the reaction mixture was refluxed for 4 h. After cooling down to room temperature, the solid was collected by filtration, washed with MeOH, and dried in air. The product was obtained as a yellow solid (230 mg, 62% yield) without further purification.

mp 353-355 °C. ^1^H NMR (300.13 MHz, Acetone-*d_6_*, 25 ºC): δ = 9.81 (s, 1H, C*H*N), 8.85 (d, ^4^*J*_H-H_ 3.0 Hz, 1H, aromatic C*H*), 8.53 (dd, ^3^*J*_H-H_ 9.0, ^4^*J*_H-H_ 3.0 Hz, 1H, aromatic C*H*), 8.17-8.14 (m, 2H, aromatic C*H*), 7.85 (ddd, ^3^*J*_H-H_ 5.7, ^3^*J*_H-H_ 8.1, ^4^*J*_H-H_ 1.5 Hz, 1H, aromatic C*H*), 7.65 (ddd, ^3^*J*_H-H_ 7.5, ^3^*J*_H-H_ 7.5, ^4^*J*_H-H_ 0.9 Hz, 1H, aromatic C*H*), 7.31-7.27 (m, 2H, aromatic C*H*), 7.22 (d, ^3^*J*_H-H_ 9.0 Hz, 1H, aromatic C*H*), 7.13-7.09 (m, 3H, aromatic C*H*). ^13^C NMR (75 MHz, Acetone-*d_6_*, 25ºC): δ = 162.2 (*C*=O), 161.5 (*C*=N), 157.4 (*C*-O), 153.1 (*C*-N), 140.6 (*C*-B), 135.4 (*C*quat), 134.5 (*C*-H), 132.0 (*C*-H), 131.3 (*C*-H), 131.2 (2C, *C*-H), 131.0 (*C*-H), 128.8 (*C*-H), 128.4 (2C, *C*-H, *C*quat), 125.9 (*C*quat), 121.1 (*C*-H), 120.2 (*C*-H). ESI^+^-MS *m/z* = 373.1 [M + H]^+^, 395.1 [M + Na]^+^; HRMS-ESI^+^ *m/z* for [C_20_H_13_O_5_N_2_B + H]^+^ calcd 373.0996, found 373.0981; HRMS-ESI^+^ *m/z* for [C_20_H_13_O_5_N_2_B + Na]^+^ calcd 395.0815, found 395.0809.

9,11-Dibromo-7-phenyl-5*H*,7*H*-7λ^4^,14λ^4^-benzo[*d*]benzo[5,6][1,3,2]oxazaborinino[2,3-*b*][1,3,2]oxazaborinin-5-one **1c**

4,6-Dibromosalicylaldehyde (1 equiv, 0.25 mmol, 70 mg) was dissolved in MeOH (20 mL). Anthranilic acid (1 equiv, 0.25 mmol, 34 mg) was added, followed by phenylboronic acid (1 equiv, 0.25 mmol, 31 mg), and the reaction mixture was refluxed for 2 h. After cooling down to room temperature, the solid was collected by filtration, washed with MeOH, and dried in air. The product was obtained as a yellow solid (74 mg, 61% yield) without further purification.

mp 353-355 °C. ^1^H NMR (300.13 MHz, Acetone-*d_6_*, 25 ºC): δ = 9.60 (s, 1H, C*H*N), 8.17-8.12 (m, 3H, aromatic C*H*), 8.05 (bs, 1H, aromatic C*H*), 7.85 (dd, ^3^*J*_H-H_ 7.7, ^3^*J*_H-H_ 7.7 Hz, 1H, aromatic C*H*), 7.66 (dd, ^3^*J*_H-H_ 7.4, ^3^*J*_H-H_ 7.4 Hz, 1H, aromatic C*H*), 7.25 (bs, 2H, aromatic C*H*), 7.10 (br s, 3H, aromatic C*H*). ^13^C NMR (75 MHz, Acetone-*d_6_*, 25ºC): δ = 161.4 (*C*=O), 161.3 (*C*=N), 155.9 (2C, *C*-O, *C*-N), 144.2 (*C*-H), 140.5 (*C*-B), 135.9 (*C*-H), 135.4 (*C*-H), 132.0 (*C*-H), 131.3 (*C*-H), 128.6 (2C, *C*-H, *C*-H), 128.3 (*C*-H), 125.8 (*C*-H), 120.1 (*C*quat), 119.6 (*C*quat), 114.6 (*C*-Br), 111.3 (*C*-Br). ESI^+^-MS *m/z* = 483.9, 485.9, 487.9 [M + H]^+^, 505.9, 507.9, 507.9 [M + Na]^+^; HRMS-ESI^+^ *m/z* for [C_20_H_12_O_3_NBBr_2_ + H]^+^ calcd 485.9335, found 485.9319.

10,12-Dihydroxy-7-phenyl-5*H*,7*H*-7λ^4^,14λ^4^-benzo[*d*]benzo[5,6][1,3,2]oxazaborinino[2,3-*b*][1,3,2]oxazaborinin-5-one **1d**

2,4,6-Trihydroxybenzaldehyde (1 equiv, 0.5 mmol, 77 mg) was dissolved in MeOH (20 mL). Anthranilic acid (1 equiv, 0.5 mmol, 69 mg) was added, followed by phenylboronic acid (1 equiv, 0.5 mmol, 61 mg), and the reaction mixture was refluxed for 2 h. After cooling down to room temperature, the solid is collected by filtration, washed with MeOH, and dried in air. After silica gel flash column chromatography (eluent: DCM/MeOH, 90/10), the product was obtained as a yellow solid (72 mg, 40% yield). The compound gave single crystals suitable for X-ray diffraction by slow evaporation from a saturated solution in MeOH.

mp > 350 °C. ^1^H NMR (300.13 MHz, Acetone-*d_6_*, 25 ºC): δ = 9.10 (s, 1H, C*H*N), 8.02 (d, ^3^*J*_H-H_ 8.0 Hz, 1H, aromatic C*H*), 7.96 (d, ^3^*J*_H-H_ 8.0 Hz, 1H, aromatic C*H*), 7.71 (dd, ^3^*J*_H-H_ 7.8, ^3^*J*_H-H_ 7.8 Hz, 1H, aromatic C*H*), 7.42 (dd, ^3^*J*_H-H_ 7.2, ^3^*J*_H-H_ 7.2 Hz, 1H, aromatic C*H*), 7.16-7.06 (m, 5H, aromatic C*H*), 5.92 (d, ^4^*J*_H-H_ 1.8 Hz, 1H, aromatic C*H*), 5.80 (d, ^4^*J*_H-H_ 1.8 Hz, 1H, aromatic C*H*). ^13^C NMR (75 MHz, Acetone-*d_6_*, 25ºC): δ = 170.6 (*C*=O), 162.7 (*C*=N), 161.8 (*C*-O), 161.0 (*C*-O), 152.5 (*C*-O), 150.7 (*C*-N), 140.5 (*C*-B), 134.5 (*C*-H), 130.4 (*C*-H), 130.2 (*C*-H), 127.6 (*C*-H), 127.3 (*C*-H), 127.2 (*C*-H), 122.7 (*C*-H), 118.5 (*C*quat), 101.8 (*C*quat), 95.8 (*C*-H), 94.6 (*C*-H). ESI^+^-MS *m/z* = 360.1 [M + H]^+^, 382.1 [M + Na]^+^; HRMS-ESI^+^ *m/z* for [C_20_H_14_O_5_NB + H]^+^ calcd 360.1043, found 360.1035.

10,12-Dimethoxy-7-phenyl-5*H*,7*H*-7λ^4^,14λ^4^-benzo[*d*]benzo[5,6][1,3,2]oxazaborinino[2,3-*b*][1,3,2]oxazaborinin-5-one **1e**

3,5-Dimethoxysalicylaldehyde (ref: *Chemical & pharmaceutical Bulletin* **1986**, *34*, 2369-2374) (1 equiv, 1 mmol, 182 mg) was dissolved in MeOH (20 mL). Anthranilic acid (1 equiv, 1 mmol, 137 mg) was added, followed by phenylboronic acid (1 equiv, 1 mmol, 122 mg), and the reaction mixture was refluxed for 4 h. After cooling down to room temperature, the solid was collected by filtration, washed with MeOH, and dried in air. The product was obtained as a yellow solid (293 mg, 76% yield) without further purification.

mp 251-253 °C. ^1^H NMR (300.13 MHz, Acetone-*d_6_*, 25 ºC): δ = 9.24 (s, 1H, C*H*N), 8.07 (dd, ^3^*J*_H-H_ 7.8, ^4^*J*_H-H_ 1.5 Hz, 1H, aromatic C*H*), 8.04 (d, ^3^*J*_H-H_ 8.4 Hz, 1H, aromatic C*H*), 7.73 (ddd, ^3^*J*_H-H_ 7.2, ^3^*J*_H-H_ 8.1, ^4^*J*_H-H_ 1.5 Hz, 1H, aromatic C*H*), 7.47 (ddd, ^3^*J*_H-H_ 7.8, ^3^*J*_H-H_ 7.8, ^4^*J*_H-H_ 1.0 Hz, 1H, aromatic C*H*), 7.29-7.26 (m, 2H, aromatic C*H*), 7.10-7.07 (m, 3H, aromatic C*H*), 6.14 (s, 2H, aromatic C*H*), 3.94 (s, 3H, OC*H*_3_), 4.00 (s, 3H, OC*H*_3_). ^13^C NMR (75 MHz, Acetone-*d_6_*, 25ºC): δ = 172.6 (*C*-OCH_3_), 163.5 (*C*-OCH_3_), 153.8 (*C*=N), 150.8 (*C*=O), 146.4 (*C*-O), 142.0 (*C*-N), 141.7 (*C*-B), 135.0 (*C*-H), 131.8 (*C*-H), 131.4 (*C*-H), 128.9 (*C*-H), 128.1 (*C*-H), 125.1 (*C*-H), 119.2 (*C*-H), 108.7 (*C*quat), 103.7 (*C*quat), 95.5 (*C*-H), 92.2 (*C*-H), 56.9 (O*C*H_3_), 56.8 (O*C*H_3_). ESI^+^-MS *m/z* = 388.1 [M + H]^+^, 410.1 [M + Na]^+^; HRMS-ESI^+^ *m/z* for [C_22_H_18_O_5_NB + H]^+^ calcd 388.1356, found 388.1351.

10-(Diethylamino)-7-phenyl-5*H*,7*H*-7λ^4^,14λ^4^-benzo[*d*]benzo[5,6][1,3,2]oxazaborinino[2,3-*b*][1,3,2]oxazaborinin-5-one **1f**

4-Diethylaminosalicylaldehyde (1 equiv, 0.5 mmol, 96 mg) was dissolved in MeOH (10 mL). Anthranilic acid (1 equiv, 0.5 mmol, 69 mg) was added, followed by phenylboronic acid (1 equiv, 0.5 mmol, 61 mg), and the reaction mixture was refluxed for 1 h. After cooling down to room temperature, the solid was collected by filtration, washed with MeOH, and dried in air. The product was obtained as a yellow solid (190 mg, 95% yield) without further purification.

mp 284-286 °C. ^1^H NMR (300.13 MHz, Acetone-*d_6_*, 25 ºC): δ = 8.98 (s, 1H, C*H*N), 8.04 (dd, ^3^*J*_H-H_ 7.8, ^4^*J*_H-H_ 1.2 Hz, 1H, aromatic C*H*), 7.90 (d, ^3^*J*_H-H_ 9.0 Hz, 1H, aromatic C*H*), 7.66 (ddd, ^3^*J*_H-H_ 7.2, ^3^*J*_H-H_ 8.1, ^4^*J*_H-H_ 1.5 Hz, 1H, aromatic C*H*), 7.49 (d, ^3^*J*_H-H_ 9.3 Hz, 1H, aromatic C*H*), 7.36 (ddd, ^3^*J*_H-H_ 7.8, ^3^*J*_H-H_ 7.8, ^4^*J*_H-H_ 1.0 Hz, 1H, aromatic C*H*), 7.29-7.26 (m, 2H, aromatic C*H*), 7.10-7.03 (m, 3H, aromatic C*H*), 6.54 (dd, ^3^*J*_H-H_ 9.3, ^4^*J*_H-H_ 2.4 Hz, 1H, aromatic C*H*), 6.13 (d, ^4^*J*_H-H_ 2.1 Hz, 1H, aromatic C*H*), 3.57 (q, ^3^*J*_H-H_ 7.2 Hz, 4H, NC*H*_2_), 1.23 (t, ^3^*J*_H-H_ 7.2 Hz, 6H, C*H*_3_). ^13^C NMR (75 MHz, Acetone-*d_6_*, 25ºC): δ = 162.9 (*C*=O), 162.8 (*C*-O), 158.2 (*C*-N), 154.8 (*C*=N), 142.3 (*C*-N), 141.7 (*C*-B), 136.2 (*C*-H), 134.7 (*C*-H), 131.8 (*C*-H), 131.4 (*C*-H), 128.0 (*C*-H), 127.8 (*C*-H), 127.5 (*C*-H), 124.6 (*C*quat), 118.2 (*C*-H), 108.5 (*C*quat), 107.8 (*C*-H), 98.4 (*C*-H), 45.7 (*C*H_2_CH_3_), 13.0 (CH_2_*C*H_3_). ESI^+^-MS *m/z* = 399.2 [M + H]^+^, 421.2 [M + Na]^+^; HRMS-ESI^+^ *m/z* for [C_24_H_23_O_3_N_2_B + H]^+^ calcd 399.1880, found 399.1869; HRMS-ESI^+^ *m/z* for [C_24_H_23_O_3_N_2_B + Na]^+^ calcd 421.1699, found 421.1689.

(*E*)-11-[(4-Methoxyphenyl)diazenyl]-7-phenyl-5*H*,7*H*-7λ^4^,14λ^4^-benzo[*d*]benzo[5,6][1,3,2]oxazaborinino[2,3-*b*][1,3,2]oxazaborinin-5-one **1g**

(*E*)-2-Hydroxy-5-[(4-methoxyphenyl)diazenyl]benzaldehyde (reference: *Talanta* **2011**, *85*, 2673-2680) (1 equiv, 0.25 mmol, 64 mg) was dissolved in MeOH (20 mL). Anthranilic acid (1 equiv, 0.25 mmol, 34 mg) was added, followed by phenylboronic acid (1 equiv, 0.25 mmol, 31 mg), and the reaction mixture was refluxed for 5 h. After cooling down to room temperature, the solid was collected by filtration, washed with MeOH, and dried in air. The product was obtained as a yellow solid (71 mg, 62% yield) without further purification.

mp 282-284 °C. ^1^H NMR (300.13 MHz, Acetone-*d_6_*, 25 ºC): δ = 9.74 (s, 1H, C*H*N), 8.39 (d, ^4^*J*_H-H_ 2.5 Hz, 1H, aromatic C*H*), 8.28 (dd, ^3^*J*_H-H_ 9.0, ^4^*J*_H-H_ 2.5 Hz, 1H, aromatic C*H*), 8.20-8.13 (m, 2H, aromatic C*H*), 7.92 (d, ^3^*J*_H-H_ 9.0 Hz, 2H, aromatic C*H*), 7.83 (ddd, ^3^*J*_H-H_ 8.2, ^3^*J*_H-H_ 7.4, ^4^*J*_H-H_ 1.6 Hz, 1H, aromatic C*H*), 7.62 (ddd, ^3^*J*_H-H_ 7.6, ^3^*J*_H-H_ 7.6, ^4^*J*_H-H_ 1.1 Hz, 1H, aromatic C*H*), 7.34-7.28 (m, 2H, aromatic C*H*), 7.19 (d, , ^3^*J*_H-H_ 9.0 Hz, 1H, aromatic C*H*), 7.16-7.08 (m, 5H, aromatic C*H*), 3.92 (s, 3H, OC*H*_3_). ^13^C NMR (75 MHz, Acetone-*d_6_*, 25ºC): δ = 163.3 (*C*=O), 162.3 (*C*=N), 162.1 (*C*-O), 161.8 (*C*-O), 147.5 (*C*-N), 146.7 (*C*-N), 140.9 (*C*-B), 135.3 (*C*-N), 133.7 (*C*-H), 131.9 (*C*-H), 131.3 (*C*-H), 130.7 (*C*-H), 129.6 (*C*-H), 128.5 (*C*-H), 128.3 (*C*-H), 125.8 (*C*quat), 125.4 (*C*-H), 123.7 (*C*quat), 120.9 (*C*-H), 120.1 (*C*-H), 115.3 (*C*-H), 114.9 (*C*-H), 56.1 (O*C*H_3_). ESI^+^-MS *m/z* = 462.2 [M + H]^+^, 484.1 [M + Na]^+^; HRMS-ESI^+^ *m/z* for [C_27_H_20_O_4_N_3_B + H]^+^ calcd 462.1625, found 462.1609; HRMS-ESI^+^ *m/z* for [C_27_H_20_O_4_N_3_B + Na]^+^ calcd 484.1445, found 484.1429.

# NMR spectra

Figure S1. ^1^H-NMR of compound **1a** in CDCl_3_.

Figure S2. ^13^C-NMR of compound **1a** in CDCl_3_.

Figure S3. ^1^H-NMR of compound **1b** in Acetone-*d*_6_.

Figure S4. ^13^C-NMR of compound **1b** in Acetone-*d*_6_.

Figure S5. ^1^H-NMR of compound **1c** in Acetone-*d*_6_.

Figure S6. ^13^C-NMR of compound **1c** in Acetone-*d*_6_.

Figure S7. ^1^H-NMR of compound **1d** in Acetone-*d*_6_.

Figure S8. ^13^C-NMR of compound **1d** in Acetone-*d*_6_.

Figure S9. ^1^H-NMR of compound **1e** in Acetone-*d*_6_.

Figure S10. ^13^C-NMR of compound **1e** in Acetone-*d*_6_.

Figure S11. ^1^H-NMR of compound **1f** in Acetone-*d*_6_.

Figure S12. ^13^C-NMR of compound **1f** in Acetone-*d*_6_.

Figure S13. ^1^H-NMR of compound **1g** in Acetone-*d_6_*.

Figure S14. ^13^C-NMR of compound **1g** in Acetone-*d_6_*.

# Details of the single-crystals XRD

Single-crystals with flake or needle shape of compounds **1a**, **1d** and **1e** were manually selected from the crystallization vial. A suitable single-crystal was mounted on a glass fiber with the help of silicon grease. Data were collected at 180(2) K on a Bruker X8 Kappa APEX II charge-coupled device (CCD) area-detector diffractometer (Mo K_a_ graphite-monochromated radiation, λ = 0.71073 Å) controlled by the APEX2 software package,^[[2]](#endnote-2)^ and equipped with an Oxford Cryosystems Series 700 cryostream monitored remotely using the software interface Cryopad.^[[3]](#endnote-3)^ Images were processed using the software package SAINT+,^[[4]](#endnote-4)^ and data were corrected for absorption by the multi-scan semi-empirical method implemented in SADABS.^[[5]](#endnote-5)^ The structure was solved using the direct methods algorithm implemented in SHELXS-97,^[[6]](#endnote-6),^^[[7]](#endnote-7)^ which allowed the immediate location of the majority of the atoms. All remaining non-hydrogen atoms were located from difference Fourier maps calculated from successive full-matrix least squares refinement cycles on *F^2^* using SHELXL-97.6^,^^[[8]](#endnote-8)^ All non-hydrogen atoms were successfully refined using anisotropic displacement parameters.

Hydrogen atoms bound to carbon were located at their idealized positions using appropriate *HFIX* instructions in SHELXL (*43* for the aromatic and vinylic, *23* for the –CH_2_– moieties and *13* for the chiral tertiary carbon atoms) and included in subsequent refinement cycles in riding-motion approximation with isotropic thermal displacements parameters (*U*_iso_) fixed at 1.2 times *U_eq_* of the atom to which they are attached.

Crystallographic data for the structures reported in this paper have been deposited with the Cambridge Crystallographic Data Centre as supplementary publication No. CCDC 2046453-2046455. Copies of the data can be obtained free of charge on application to CCDC, 12 Union Road, Cambridge CB2 2EZ, U.K. Fax: (+44) 1223 336033. E-mail: deposit@ccdc.cam.ac.uk.

# Absorption and emission spectra

Figure S15. Absorption and emission spectra of compound **1a** in tetrahydrofuran.

Figure S16. Absorption and emission spectra of compound **1b** in tetrahydrofuran.

Figure S17. Absorption and emission spectra of compound **1c** in tetrahydrofuran.

Figure S18. Absorption and emission spectra of compound **1d** in tetrahydrofuran.

Figure S19. Absorption and emission spectra of compound **1e** in tetrahydrofuran.

Figure S20. Absorption and emission spectra of compound **1f** in tetrahydrofuran.

Figure S21. Absorption and emission spectra of compound **1g** in tetrahydrofuran.


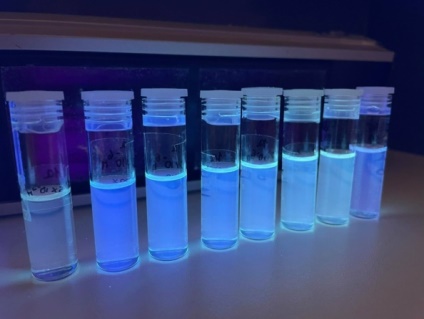


Figure S22. Emission spectra of compound **1a** in THF-water solvents (left) and relative intensities (right). Structure of the complex and photographs of the solutions/suspensions under a hand-held UV lamp (365nm).


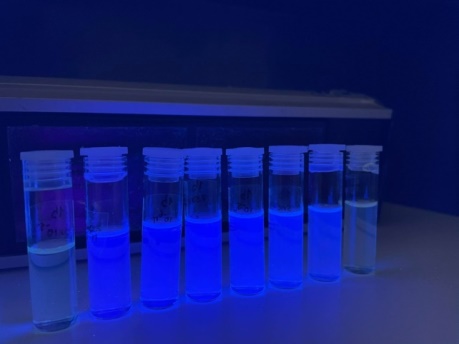


Figure S23. Emission spectra of compound **1b** in THF-water solvents (left) and relative intensities (right). Structure of the complex and photographs of the solutions/suspensions under a hand-held UV lamp (365nm).


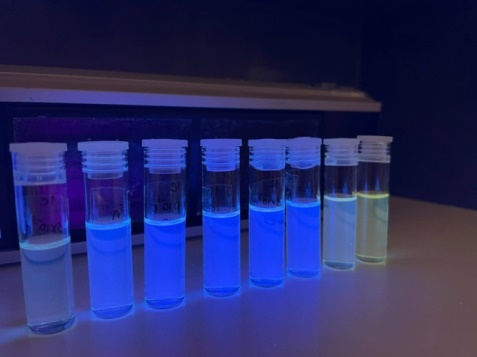


Figure S24. Emission spectra of compound **1c** in THF-water solvents (left) and relative intensities (right). Structure of the complex and photographs of the solutions/suspensions under a hand-held UV lamp (365nm).


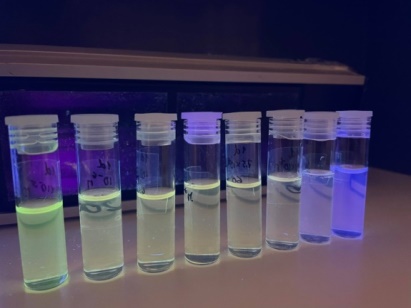


Figure S25. Emission spectra of compound **1d** in THF-water solvents (left) and relative intensities (right). Structure of the complex and photographs of the solutions/suspensions under a hand-held UV lamp (365nm).


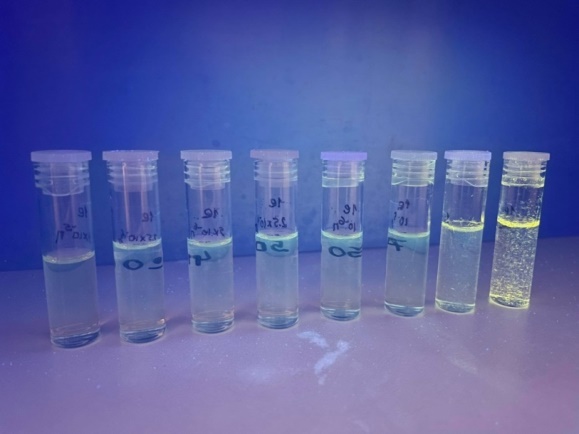


Figure S26. Emission spectra of compound **1e** in THF-water solvents (left) and relative intensities (right). Structure of the complex and photographs of the solutions/suspensions under a hand-held UV lamp (365nm).


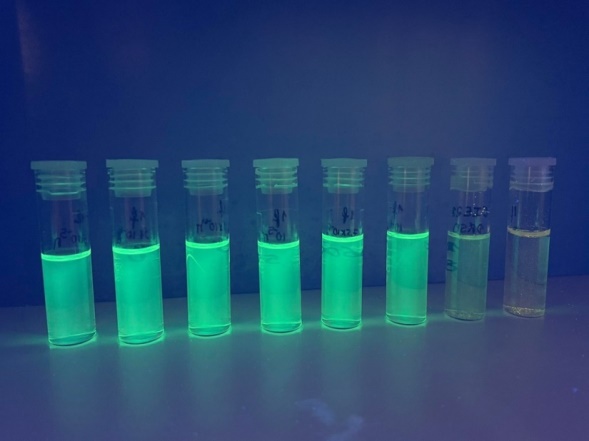


Figure S27. Emission spectra of compound **1f** in THF-water solvents (left) and relative intensities (right). Structure of the complex and photographs of the solutions/suspensions under a hand-held UV lamp (365nm).


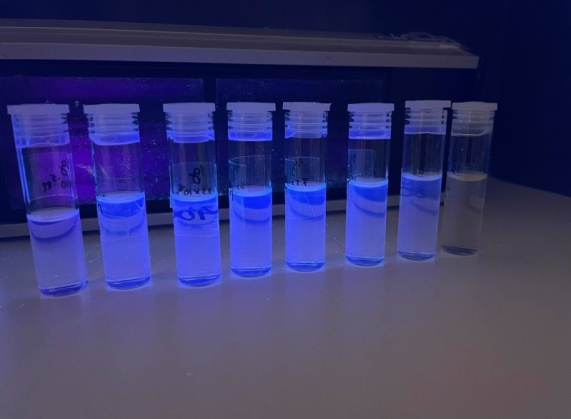


Figure S28. Emission spectra of compound **1g** in THF-water solvents (left) and relative intensities (right). Structure of the complex and photographs of the solutions/suspensions under a hand-held UV lamp (365nm).

# Bibliography

1. M. Adib, E. Sheikhi, H. R. Bijanzadeh, L.-G. Zhu, *Tetrahedron* **2012**, *68*, 3377-3383. [↑](#endnote-ref-1)
2. APEX2, Data Collection Software Version 2.1-RC13, Bruker AXS, Delft, The Netherlands 2006. [↑](#endnote-ref-2)
3. Cryopad, Remote monitoring and control, Version 1.451, Oxford Cryosystems, Oxford, United Kingdom 2006. [↑](#endnote-ref-3)
4. SAINT+, Data Integration Engine v. 7.23a© 1997-2005, Bruker AXS, Madison, Wisconsin, USA. [↑](#endnote-ref-4)
5. G. M. Sheldrick, SADABS v.2.01, Bruker/Siemens Area Detector Absorption Correction Program 1998, Bruker AXS, Madison, Wisconsin, USA. [↑](#endnote-ref-5)
6. G. M. Sheldrick, *Acta Cryst. A*, **2008**, *6*4, 112-122. [↑](#endnote-ref-6)
7. G. M. Sheldrick, SHELXS-97, Program for Crystal Structure Solution, University of Göttingen 1997. [↑](#endnote-ref-7)
8. G. M. Sheldrick, SHELXL-97, Program for Crystal Structure Refinement, University of Göttingen 1997. [↑](#endnote-ref-8)
